# EfpA is required for regrowth of *Mycobacterium tuberculosis* following isoniazid exposure

**DOI:** 10.1128/aac.00261-24

**Published:** 2024-07-22

**Authors:** Adam H. Roberts, Christopher W. Moon, Valwynne Faulkner, Sharon L. Kendall, Simon J. Waddell, Joanna Bacon

**Affiliations:** 1 Discovery Group, VDEC, UK Health Security Agency, Porton Down, Salisbury, United Kingdom; 2 Global Health and Infection, Brighton and Sussex Medical School, University of Sussex, Brighton, United Kingdom; 3 Centre for Emerging, Endemic and Exotic Diseases, Pathobiology and Population Sciences, Royal Veterinary College, Hatfield, United Kingdom; St. George's, University of London, London, United Kingdom

**Keywords:** EfpA, efflux, CRISPRi, *Mycobacterium tuberculosis*, TB, isoniazid, regrowth, antibiotic resistance, early bactericidal activity

## Abstract

Efflux of antibiotics is an important survival strategy in bacteria. *Mycobacterium tuberculosis* has approximately sixty efflux pumps, but little is known about the role of each pump or the substrates they efflux. The putative efflux pump, EfpA, is a member of the major facilitator superfamily and has been shown to be essential by saturation transposon mutagenesis studies. It has been implicated in the efflux of isoniazid (INH), which is a first-line drug used to treat tuberculosis (TB). This is supported by evidence from transcriptional profiling showing that *efpA* is induced in response to INH exposure. However, its roles in the physiology and adaptation of *M. tuberculosis* to antibiotics have yet to be determined. In this study, we describe the repression of *efpA* in *M. tuberculosis,* using CRISPR interference (CRISPRi) to knockdown the expression of this essential gene and the direct effect of this on the ability of *M. tuberculosis* to survive exposure to INH over a 45-day time course. We determined that wild-type levels of *efpA* were required for recovery of *M. tuberculosis* following INH exposure and that, after 45 days of INH exposure, only a few viable colonies were recoverable from *efpA*-repressed *M. tuberculosis*. We conclude that EfpA is required for recovery of *M. tuberculosis* following INH exposure, which could reduce the efficacy of INH *in vivo*, and that EfpA may have a role in the development of resistance during drug therapy.

## INTRODUCTION

Infection with *Mycobacterium tuberculosis* is the leading cause of death from a single infectious agent. In 2022, approximately 10.6 million people became ill due to tuberculosis (TB) and 1.3 million people died from TB worldwide (1). Multidrug resistance in *M. tuberculosis*, defined as resistance to at least isoniazid (INH) and rifampicin, has also compounded the burden of TB infections globally, with 410,000 cases estimated to have occurred in 2022 alone (1). The lengthy and toxic nature of TB multidrug therapy highlights the need for improved regimens with lower toxicity and shortened treatment times. This requires greater insights into new and existing mycobacterial drug targets for which promising new therapies will emerge to combat the global TB burden.

Although drug resistance in *M. tuberculosis* is predominantly caused by alterations in drug-activating enzymes or drug targets through DNA mutation, there is increasing evidence of enhanced expression of efflux pumps (including EfpA) in multidrug-resistant clinical isolates (2–4), which may contribute to the development of drug resistance. Studies by Machado et al. in 2012 (5) and Rodrigues et al. in 2012 (6) demonstrated a relationship between the overexpression of genes encoding efflux pumps and increased efflux activity, with an increased frequency in *katG* mutations. This supports the hypothesis that efflux of an antibiotic provides a window of opportunity for genetically encoded drug resistance to emerge. Therefore, the discovery of molecules that inhibit the action of efflux pumps, potentially extending the life of current and new therapies, would be an advantage in combating both drug-susceptible and drug-resistant *M. tuberculosis* infections (7, 8).

The efflux pump, EfpA, which belongs to the major facilitator family, is essential in *M. tuberculosis,* as demonstrated by transposon mutagenesis studies (9–12). This essentiality brings challenges as the function cannot be studied by deleting the gene. The first study to identify and characterize the gene, *efpA*, was by Doran et al. in 1997 (13), who found that *efpA* encodes a putative efflux protein that is predicted to be highly related to the QacA transporter family, with orthologs in *M. marinum, M. leprae, M. smegmatis,* and *M. bovis*. It is possible that the differential regulation of *efpA* impacts the survival of *M. tuberculosis* in the acidic compartment of the macrophage as its expression is reduced under acidic conditions (14). Transcriptomics studies also revealed that in *M. tuberculosis*, *efpA* is more highly expressed during INH exposure *in vitro* (15–19) and in sputa (20).

Populations of *M. tuberculosis* exposed to INH, *in vitro*, exhibit a substantial loss in bacterial viability during the first 2 days followed by a period of regrowth (19, 21–23), which is not entirely explained by an increase in the INH-resistant mutant frequency (19). This biphasic response has also been observed in *M. tuberculosis*-infected guinea pigs treated with INH and was found to be associated with the emergence of antibiotic-tolerant persisting populations that were not INH-resistant mutants (24). Efflux has been suggested as a mechanism that might contribute to *M. tuberculosis* drug tolerance and development of resistance (25).

There is direct evidence for the role of *efpA* in the response of *M. tuberculosis* to the standard drug regimen during the early stages of treatment; *efpA* was induced in *M. tuberculosis* derived from patient sputa 3 days after the start of drug therapy (20). Understanding the contributions of efflux to drug tolerance and emergence of genotypic resistance will drive the development of novel efflux-targeting therapeutics. More specifically, this study aims to help define the role of EfpA, in mediating the efficacy of the frontline drug INH. Since *efpA* is an essential gene, we used CRISPRi knockdown to determine the functional significance of EfpA in *M. tuberculosis* after exposure to INH.

## MATERIALS AND METHODS

### Bacterial strains, plasmids, and culture conditions

*M. tuberculosis* H37Rv strains were grown in Difco Middlebrook 7H9 medium (Sigma-Aldrich), supplemented with 0.5% glycerol (VWR), 0.2% Tween-80 (Sigma-Aldrich), and 10% oleic acid dextrose catalase (OADC) growth enrichment supplement (UKHSA, Porton Down Media services). Middlebrook 7H10 medium was supplemented with 0.5% glycerol and 10% OADC. Strains containing plasmids pRH2521 and pRH2502 were selected for in culture using kanamycin (25 µg mL^−1^) and hygromycin (50 µg mL^−1^). All liquid cultures were grown at 37°C, with shaking at 200 rpm. Each strain was grown from frozen stocks for 10 days and standardized to an optical density of 0.05 OD_540nm_ for the inoculation of time course experiments.

### Antibiotic stock production

INH (Sigma-Aldrich) and anhydrotetracycline (ATc) (Sigma-Aldrich) liquid stocks were prepared in dimethyl sulfoxide (DMSO), whereas kanamycin (Sigma-Aldrich) and hygromycin (Sigma-Aldrich) liquid stocks were prepared in water. All drug stocks were filter-sterilized using a 0.2-µm filter (Sartorius Stedim).

### Plasmid and strain construction

The extrachromosomal pRH2521 plasmid, encoding for single-guide RNA (sgRNA) scaffold sequences, and the integrative pRH2502 plasmid that encoded for a deactivated Cas9 (dCas9) from *Streptococcus pyogenes* (Table 1) (both under the control of Tet-regulatory promoters) were used for *efpA* repression. The two-plasmid CRISPRi system was developed for *M. tuberculosis* by Singh et al. in 2016 (26), and the plasmids pRH2521 and pRH2502 were supplied by Addgene (Addgene plasmid # 84380 and Addgene plasmid # 84379). The *efpA*-targeting sequence was cloned into the sgRNA (26–29). Initially, a 20-nucleotide DNA sequence that is 98 nucleotides downstream of the start of the *efpA* open-reading frame (ORF) was identified as a target for sgRNA base-pairing. An *efpA-*targeting sgRNA pRH2521 plasmid was constructed (27), and this plasmid (1 µg) was electroporated into an electrocompetent *M. tuberculosis* H37Rv_dCas9_ strain (containing pRH2502 plasmid) using a Gene Pulser Xcell (Bio-Rad) and following a published method (30). A pulse of 2.5 kV, 25 µF, and a resistance setting of 1,000 Ω were used. The successful transformants were selected on Middlebrook 7H10 medium containing 25 µg mL^−1^ of kanamycin and 50 µg mL^−1^ of hygromycin. The *M. tuberculosis* H37Rv strain containing pRH2521 + *efpA*-targeting sgRNA (referred to as “*efpA*^KD^”) was compared to a control strain generated using the same approach, except for a sgRNA with a random base-pairing sequence that was not specific to any *M. tuberculosis* gene target (referred to as “control strain”).

**TABLE 1:** Strains and plasmids used

Strain or plasmid	Description	Source
**Strains**		
*E. coli* DH5α	High transformation efficiency competent cells (T1 phage resistant and *endA* deficient)	New England Biolabs
*M. tuberculosis* H37Rv	*M. tuberculosis* laboratory research reference strain	(31)
*M. tuberculosis* H37Rv_dCas9_	*M. tuberculosis* H37Rv containing the pRH2502 integrative plasmid encoding *Streptococcus pyogenes* dCas9 and Kan^R^	(29)
*M. tuberculosis* H37Rv_dCas9_ sgRNA control (“control strain”)	*M. tuberculosis* H37Rv_dCas9_ containing the pRH2521 extrachromosomal plasmid with a randomized sgRNA sequence, Kan^R^, and Hyg^R^	(29)
*M. tuberculosis* H37Rv_dCas9_*efpA* knockdown (“*efpA*^KD^”)	*M. tuberculosis* H37Rv_dCas9_ containing the pRH2521 extrachromosomal plasmid with an sgRNA sequence targeting *efpA* at +98 + 120 bp downstream of start of *efpA* open reading frame, Kan^R^, and Hyg^R^	This study
**Plasmids**		
pRH2502	Integrative plasmid originating from the pTC-0X-1L backbone, expressing *S. pyogenes* dCas9 under control of the tetracyline-inducible *tetRO* promoter (uv15*tetO*)	(26); pRH2502 (Addgene plasmids # 84379)
pRH2521	Extrachromosomal plasmid originating from the pTE-10M-0X backbone, expressing sgRNA (randomized base-pairing sequence) under control of the tetracyline-inducible *tetRO* promoter (P_myc1_*tetO*)	(26); pRH2521 (Addgene plasmids # 84380)
pRH2521 *efpA^KD^* +98 + 120	pRH2521 with the sgRNA base-pairing region targeting *efpA* at +98 + 120 bp downstream of start of the *efpA* open-reading frame, cloned into the plasmid	This study

### ATc induction of *efpA* repression

The *efpA*^KD^ and control strains were grown in Middlebrook 7H9 medium over 14 days in a volume of 13 mL, in universal tubes, in triplicate. At the point of inoculation, ATc was added at a final concentration of 200 ng mL^−1^. Culture samples were collected at intervals, spun at 6,000 rpm for 10 minutes, and the pellet was resuspended in phosphate-buffered saline (PBS) pH 7.4. Total viable counts (CFU mL^−1^) were determined using the Miles–Misra technique. The plates were incubated for 3 weeks at 37°C in a static incubator prior to colony enumeration.

### *M. tuberculosis* cultures exposed to INH

To assess the impact of INH exposure, the *efpA*^KD^ and control strains were grown in Middlebrook 7H9 medium over 45 days, in triplicate. At the point of inoculation, ATc (final concentration of 200 ng mL^−1^) and/or INH (final concentration of 0.5 µg mL^−1^) were added to the cultures. The time-kill experiments also included a no-drug condition. Culture samples were collected at intervals, spun at 6,000 rpm for 10 minutes, and the pellets were resuspended in phosphate-buffered saline (pH 7.4). Viability was measured using total viable counts (CFU mL^−1^), as described previously.

### RNA extraction and purification

RNA was extracted from 10 mL of culture that had been sampled at intervals of 0, 1, 3, and 7 days post ATc-addition. Forty milliliters of guanidine thiocyanate (GTC) lysis solution (comprising 5 M GTC (Sigma-Aldrich), 0.5% lauryl sarcosine (Sigma-Aldrich), 25 mM trisodium citrate (Sigma-Aldrich), 0.5% Tween-80, and 0.05% 2-mercaptoethanol (Sigma-Aldrich) was added to the culture samples and incubated for 1 hour at room temperature. The cultures were spun at 3,000 rpm for 10 minutes, the supernatant was removed, and the *M. tuberculosis* pellets were resuspended in 1.2 mL TRIzol solution (Thermo). The extractions were transferred to tubes containing lysing matrix B beads (0.5 mL of 0.1 mm) (MP bio) and disrupted in a bead beater at full power for 50 seconds. The solution was spun at 13,000 rpm for 10 minutes, resuspended in 240 µL 24:1 chloroform:isoamyl alcohol (Sigma-Aldrich), and shaken vigorously for 20 seconds. Following centrifugation at 13,000 rpm for 10 minutes, the aqueous phase was resuspended in 600 µL chloroform:isoamyl alcohol, spun again, and the step was repeated. The aqueous phase was added to 600 µL isopropanol (Sigma-Aldrich) with 60 µL sodium acetate (Sigma-Aldrich) and incubated overnight at −70°C to precipitate the nucleic acids. The sample was washed with ethanol, resuspended in nuclease-free water, and purified using a Qiagen RNeasy kit with additional on-column DNase (Qiagen) digestion. RNA quantity and purity were assessed by using a spectrophotometer (Nanodrop; Thermo).

### Quantitative reverse transcription-PCR (qRT-PCR)

The absolute quantification method was used to determine the number of transcripts (copy number) of *efpA* in each sample, relative to an endogenous housekeeping control gene, *sigA*. To achieve a copy number standard curve, full gene fragments of *efpA* and *sigA* were amplified from *M. tuberculosis* H37Rv genomic DNA using the PCR primers outlined in Table 2, GoTaq green polymerase (Promega) and DMSO (Sigma-Aldrich), using conditions of 98°C for 2 minutes, followed by 35 cycles of 94°C for 30 seconds, 59°C for 30 seconds, and 72°C for 2 minutes, with a final cycle of 72°C for 10 minutes. The PCR products were purified using a PCR clean-up kit (Qiagen), and the DNA concentration was quantified using a Qubit dsDNA broad range quantification assay (Thermo). From the quantity and gene length, the copy number of *efpA* and *sigA* was calculated using the Thermo Fisher copy number calculator. The copy number values were standardized to 10^7^ and used to generate a standard curve of *efpA* and *sigA* abundance. The reverse transcription reactions to synthesize cDNA consisted of 500 ng total RNA, 1 µL Superscript III (Thermo), 4 µL First-Strand Synthesis Buffer (Thermo), 1 µL 10 mM dNTP mixture (Thermo), and 1 µL random primers (Thermo). The reactions also featured a no reverse transcriptase control for each sample. For the qRT-PCR reactions, 10 µL Taqman Universal Master-Mix II with UNG (Thermo), 5 µL of cDNA/control DNA fragment, 1 µL of forward and reverse primers, 0.5 µL Taqman probe, and nuclease-free water were added to reach a total reaction volume of 20 µL in a 96-well MicroAmp plate (Thermo). The reaction cycling conditions used were 2 minutes at 95°C followed by 45 cycles of 95°C for 30 seconds and then 60°C for 30 seconds using the QuantStudio 7 Real-Time PCR system (Thermo). Each qRT-PCR experiment featured non-template controls. The data were analyzed using the QuantStudio Real-Time PCR Software (Thermo), and the *efpA* copy number was normalized as a ratio to the *sigA* copy number. The values from the ATc-induced *efpA*^KD^ reactions were compared to that of the ATc-induced control strain and *efpA*^KD^ without ATc induction.

**TABLE 2:** Primers and oligonucleotides used in the qRT-PCR^*a*^

Primer or oligonucleotide	Sequence (5’ to 3’)
**CRISPRi oligonucleotide & target location**	
*efpA* +98 + 120 forward	GGGAGTCGTTGAGAGCCGTCATAG
*efpA* +98 + 120 reverse	AAACCTTCAGAGCGCCCCAGCAGG
**Primers for full gene amplification**	
*efpA* full gene forward	ACTGGTGGTTCAACACGGAA
*efpA* full gene reverse	GGATCAGCAGTTTGTGGCTG
*sigA* full gene forward	GACACTTTCGGTTACGCACG
*sigA* full gene reverse	GCTGATTCGAACTCCGATCC
**qRT-PCR primers and probes**	
qRT_PCR *efpA* forward	AGGTTTCATCCCGTTCGTCA
qRT_PCR *efpA* reverse	GCCGAATAGCAGATATCCGC
qRT_PCR *efpA* probe	CCTCGCAGCTGGTGTCCCGGT
qRT_PCR *sigA* forward	CCGGTGATTTCGTCTGGGAT
qRT_PCR *sigA* reverse	ATCTGTTTGAGGTAGGCGCG
qRT_PCR *sigA* probe	ACGAGTCGGAGGCCCTGCGT

^*a*^ Underlined bases indicate the first four bases of CRISPRi oligonucleotides which are “overhang sequences” used to clone the sgRNA base-pairing sequence into pRH2521.

### Gene expression profiling

The quality of the extracted RNA was confirmed using a Bioanalyzer (Agilent Technologies). Cy3-labeled cDNA was produced by mixing 1 µg of total RNA with 1 µL of random primers (Thermo), heated to 70°C for 2 minutes, and then snap-frozen on ice. The RNA was then added to 14 µL of master-mix consisting of 15.3 µL Cy3 dCTP (Sigma-Aldrich), 20.7 µL dNTP mix (Thermo), 22.5 µL DTT (Thermo), 45 µL 5 x First-Strand Buffer (Thermo), and 22.5 µL SuperScript II (Thermo). This reaction mix was incubated in the dark at 25°C for 10 minutes, followed by 42°C for 90 minutes, after which it was purified using a MinElute PCR Purification kit (Qiagen). For hybridization, 18 µL of purified Cy3-labeled cDNA was added to 4.5 µL of 10 x blocking agent (Agilent) and 22.5 µL of 2 x hybridization buffer (Agilent). The reaction mix was incubated at 95°C for 5 minutes, followed by a 1-minute centrifugation at 13,000 rpm. Forty microliters of the sample was pipetted onto a hybridization slide (Agilent) and incubated at 65°C in a hybridization oven set to rotate at 20 rpm. Following overnight incubation, the slides were washed in Oligo aCGH wash buffer 1 (Agilent) at room temperature for 5 minutes with agitation and then in Oligo aCGH wash buffer 2 (Agilent) at 37°C for 1 minute with agitation. Slides were scanned immediately using an Agilent DNA Microarray Scanner (with SureScan 2 µm High-Resolution Technology; G2505C). The microarrays used were custom BµG@S ‘TBv3_0_0’ arrays (accession number A-BUGS-41) that were produced by Agilent Technologies.

### Gene expression analyses

Features were extracted from the array images using Agilent Feature Extraction Software (v10.7) with local background correction. The extracted data were analyzed in the RStudio software (version 1.3.1056) using the Limma package (version 3.14) available through Bioconductor (32). These data were log_2_-transformed and normalized by median absolute deviation (MAD) analysis prior to comparisons in Limma. The data were fitted to a linear model, and pairwise t-statistic comparisons were performed between the expression data for each gene between the conditions at each time point. Using empirical Bayesian statistics and the t-statistics highlighted previously, fold-changes in differential gene expression were determined between paired conditions. The outputs of the Limma analysis were the log_2_ fold changes in gene expression and the *P*-values from the t-statistics. These comparisons were further corrected for multiple testing using Benjamini–Hochberg adjustment to provide an adjusted *P*-value, considering the false discovery rate (FDR). At each timepoint, ATc-induced *efpA*^KD^ was compared to non-induced *efpA*^KD^ to define transcriptional changes associated with the repression of *efpA*. The data sets were filtered to include differentially expressed genes that exhibited an adjusted *P*-value of <0.05 and a log_2_ fold-change difference of greater or less than 1 (fold difference of greater or less than 2), detailed in Supplementary Data ”Differential_expression_efpA_knockdown _S1.” The gene functions and names were assigned using DAVID enrichment software (https://david.ncifcrf.gov/) (33) and Mycobrowser (https://mycobrowser.epfl.ch/) (34). The microarray data have been assigned ArrayExpress accession E-MTAB-13755 .

### Scanning electron microscopy

*efpA*^KD^ cultures, with or without ATc induction, were fixed after 7 days of culture, using a final concentration of 4% formaldehyde, spun by centrifugation at 6,000 rpm for 10 minutes, resuspended in water, and imaged by scanning electron microscopy (SEM) using the following method. Cells were immobilized onto glass cover slips, further fixed with osmium tetroxide for 2 hours at room temperature, followed by dehydration by exposure to graded series of ethanol. Samples were then treated with hexamethyldisilane and air-dried. A conductive coating of 20 nm gold was applied using a fine-grain ion-beam sputter coater, prior to imaging using a Zeiss Sigma 300VP Scanning Electron Microscope. To determine changes in the cell dimensions, SEM images were analyzed in ImageJ (35). The proximal widths and lengths of one hundred and eighty-seven randomly selected bacilli were measured using a calibrated drawline function, which was set to the scale bar of each image.

### Statistical methods

For the growth curves (Fig. 2), a two-way ANOVA was applied to identify significant differences in the viable count (CFU mL^−1^) across the time course between strains with or without ATc induction. For the INH time-kill experiments (Fig. 7), a three-way ANOVA was utilized to compare viability (CFU mL^−1^) between strains, ATc induction, and/or INH exposure. All viable counts were log_10_-transformed prior to the statistical analyses. A one-way ANOVA with multiple comparisons was applied to compare measurements of the cell dimensions between different conditions (strains; ATc induction). ANOVA was performed using RStudio software (version 1.3.1056).

## RESULTS

### Targeted repression of *efpA* results in a loss of viability in *M. tuberculosis*

**Fig 1:**
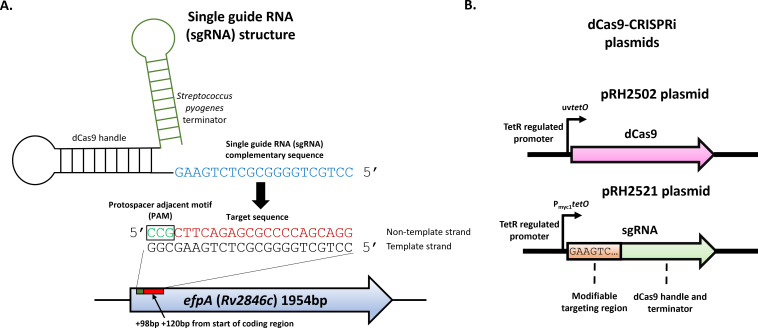
A two-plasmid dCas9–sgRNA CRISPRi system adapted to repress *efpA* expression. Schematic of the single-guide RNA chimeric structure consisting of the terminator region originating from *Streptococcus pyogenes,* the dCas9 handle region (to enable binding to the dCas9 enzyme), and the sequence complementary to the *efpA* target sequence at the 5’ end of the gene. The protospacer adjacent motif (PAM) sequence (CCN) is adjacent to the 5’ end of the target sequence (**A**). Schematic of the CRISPRi genes on two plasmids coding for the CRISPRi system: pRH2502, which encodes the dCas9 endonuclease enzyme under the control of a uv*tetO* promoter, and pRH2521-*efpA*, which encodes the *efpA* sgRNA under the control of a p*myc1tetO* promoter (**B**).

The aim of this study was to determine the role of EfpA in the response of *M. tuberculosis* to INH. We initially evaluated the effect of *efpA* repression on the viability of *M. tuberculosis* in the absence of INH. Since *efpA* is an essential gene in *M. tuberculosis*, an *efpA* knockdown strain (“*efpA*^KD^”) was generated by CRISPRi and induced with anhydrotetracycline (ATc), using the two plasmid–dCas9 system (26). The sgRNA targeted *efpA* in a 20-bp region between +98 bp to +120 bp, relative to the start codon of the *efpA* coding region, downstream of a 3-bp protospacer adjacent motif (PAM) required for dCas9 binding (Fig. 1). A control strain was generated (“control strain”) that contained the same vectors but with a random sgRNA sequence, rather than an *efpA*-targeting sgRNA, as a comparator in all experiments (26).

**Fig 2:**
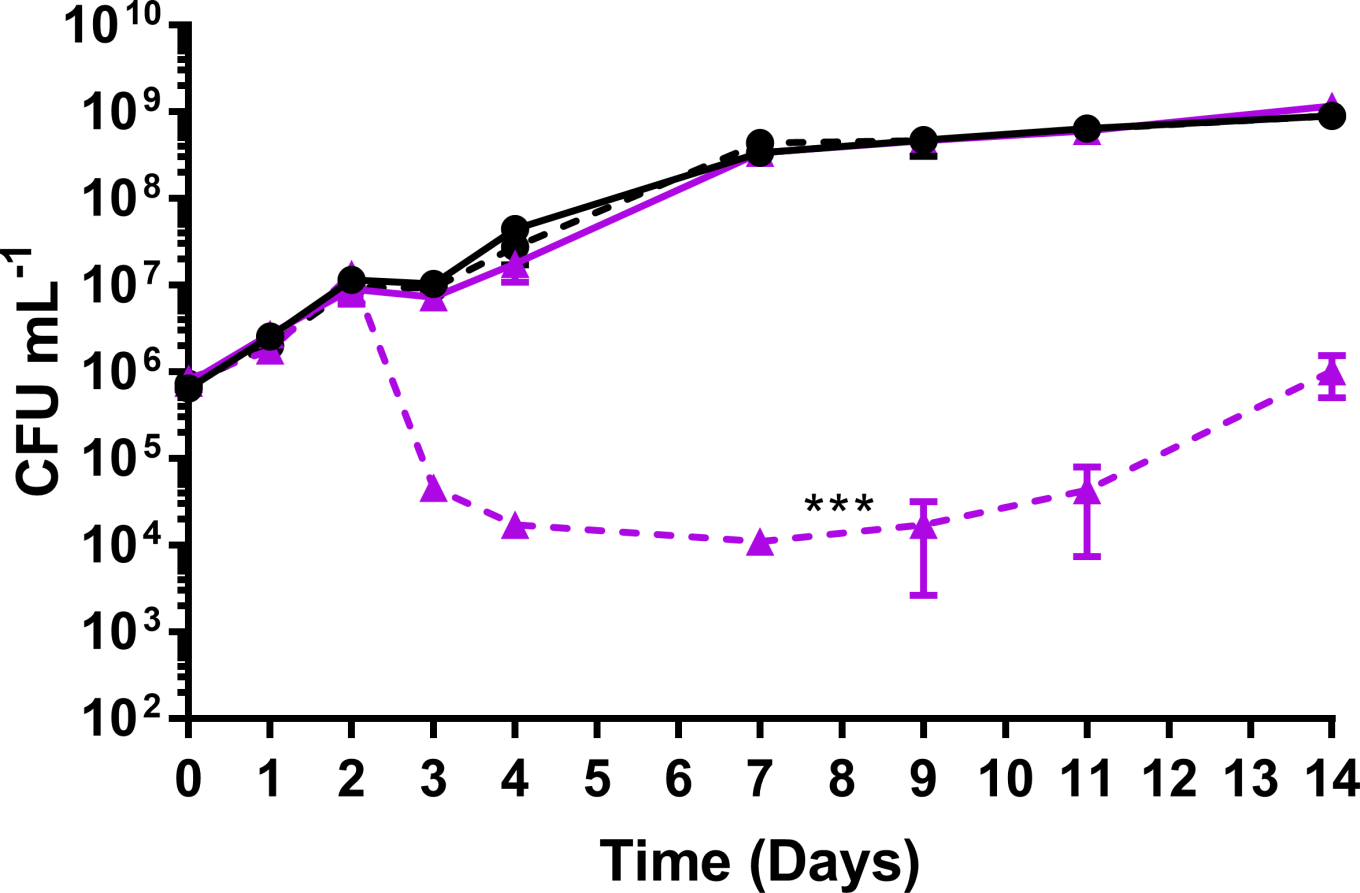
A comparison of the total viable counts (CFU mL^−1^) for the control strain (**solid line**) and *efpA*^KD^ (**dotted line**) cultured over a 14-day time course with (**purple triangles**) or without (**black circles**) 200 ng/ml ATc to induce *efpA* knockdown. The ATc was added to respective cultures on day 0. All values represent the mean of three biological replicates. Error bars are ±SD. Statistical significance was determined across the whole time course by two-way ANOVA comparing the responses of each strain with and without ATc addition. *** =*P* ≤ .001.

The viability of *efpA*^KD^ was determined after ATc induction of *efpA* repression compared to a non-induced state, in triplicate cultures, over a 14-day time course in Middlebrook 7H9 medium. ATc was added on day 0 of the time course at a final concentration of 200 ng mL^−1^. Samples were removed at days 0, 1, 2, 3, 4, 7, 9, 11, and 14, for total viable counts (Fig. 2). The conditions such as the concentration of ATc and the time of addition were optimized prior to these experiments to maximally reduce *efpA* expression.

The repression of *efpA* resulted in a steep reduction in the viability of *efpA*^KD^ between days 2 and 4 of the time course (Fig. 2), and there was a statistically significant difference between the total viable counts compared to those for non-induced *efpA*^KD^ (*P* = <0.001). Despite a gradual increase in viability from day 7, by day 14, the *efpA*^KD^ was unable to recover to the same level of viability as *efpA*^KD^ without ATc induction. As expected, ATc addition did not affect the viability of the control strain.

### Induction of the CRISPRi system targeting *efpA* leads to a dramatic reduction in *efpA* expression and changes in cell morphology

**Fig 3:**
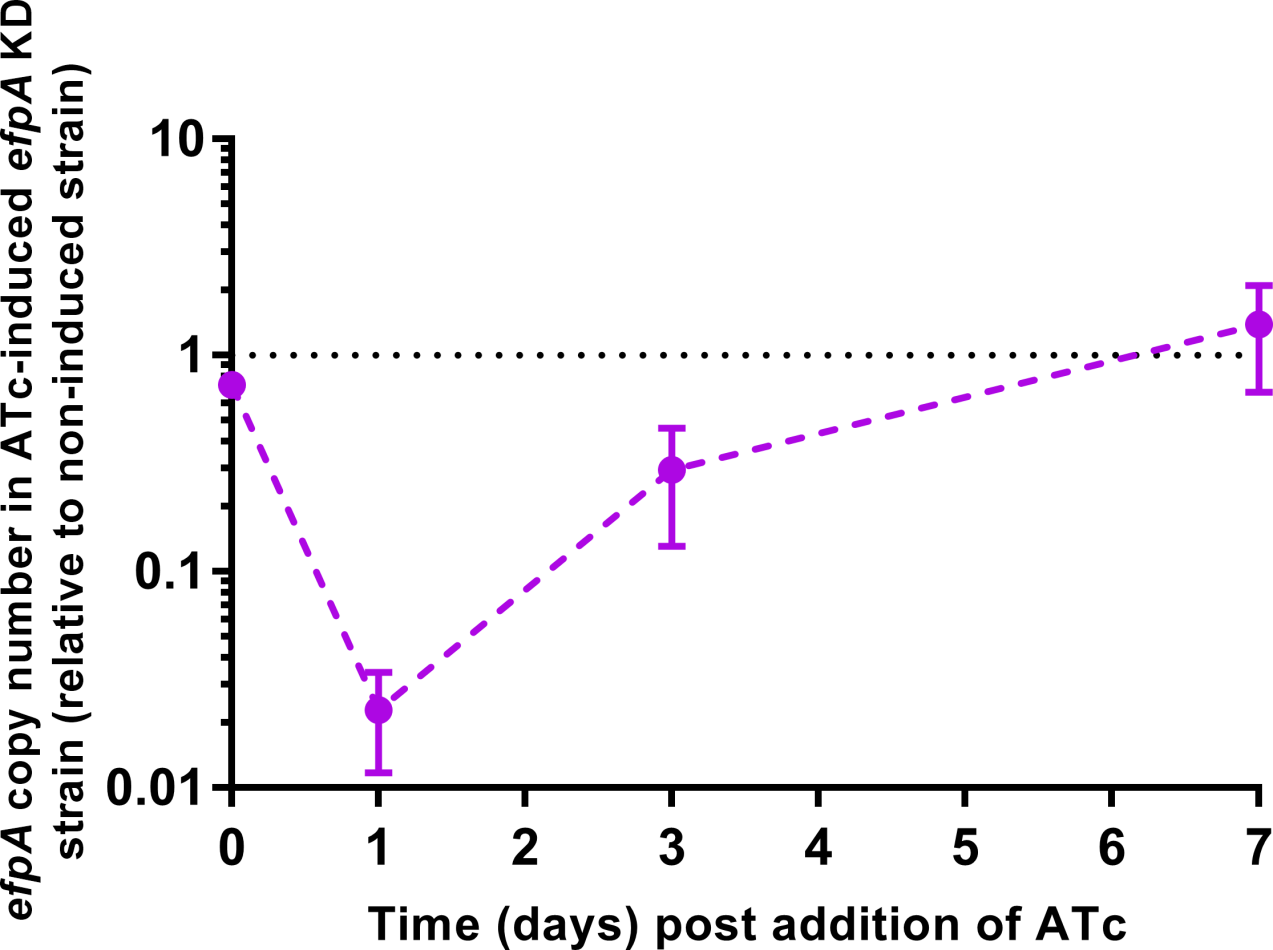
Repression of *efpA* was determined by qRT-PCR. ATc was added after 4 days of growth. *efpA* copy number values were normalized relative to *sigA* copy number values across the time course. Error bars are ±SD of the mean of three biological replicates.

To assess the knockdown of *efpA* expression, the copy number of *efpA* mRNA transcripts was determined for *efpA*^KD^ with or without ATc induction, using qRT-PCR. ATc was added in the mid-exponential phase, and *efpA* transcript copy numbers were quantified (Fig. 3). At day 1 post-induction, *efpA* transcript levels were 43-fold lower in the induced *efpA*^KD^ cultures than in the non-induced cultures, confirming the repression of *efpA* expression (Fig. 3). Following this, the transcript levels increased over time and were 3-fold lower in the induced *efpA*^KD^ by day 3 post-induction of knockdown and finally recovered to equivalent expression levels by day 7.

**Fig 4:**
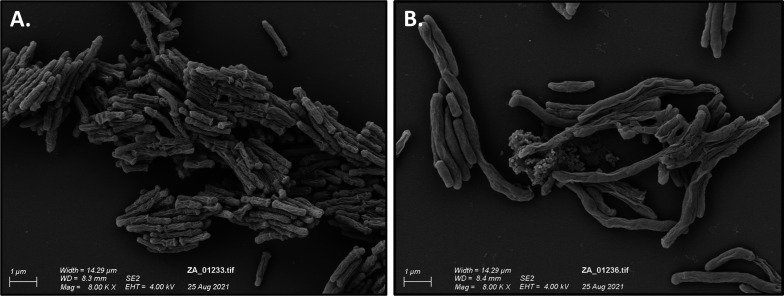
Scanning electron micrographs of *efpA*^KD^ in the absence (**A**) or presence of 200 ng ml^−1^ ATc (**B**). Scanning electron micrographs were imaged at 8,000 x magnification.

**Fig 5:**
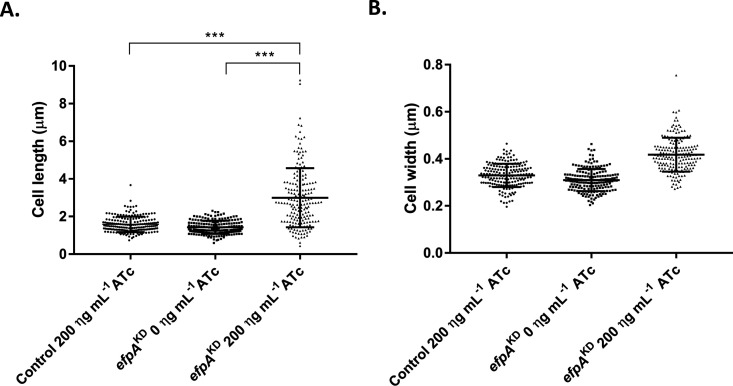
Scatter plots of the cell length (**A**) and the cell width (**B**) of either the control strain or *efpA*^KD^ in the absence or presence of 200 ng mL^−1^ ATc. The cell length and width were measured from one hundred and eighty-seven bacilli randomly selected from SEM images at 2000 x magnification. Error bars are ±SD. Statistical significance was determined between the different conditions (strains, +/-ATc) by one-way ANOVA. *** =*P* ≤ .001.

To observe whether the repression of *efpA* had an impact on cellular morphology, scanning electron microscopy (SEM) was used to study *efpA*^KD^ with or without ATc addition. Images of the culture samples were taken on day 7; this was the timepoint at which the cell morphology effects were most visible. Repression of *efpA* led to the formation of elongated bacilli (Fig. 4). We observed a statistically significant increase (*P* = <0.001) in the average cell length of ATc-induced *efpA*^KD^ (3 µm ± 1.573 SD) compared to *efpA*^KD^ without ATc (1.44 µm ± 0.355 SD) (Fig. 5). Visually, the data indicated increased cell widths with *efpA* repression, although this was not statistically significant. Normal cell morphology was restored by day 14. These results suggest the role of EfpA in cell division.

### Repression of *efpA* leads to reduced expression of cell wall functions

**Fig 6:**
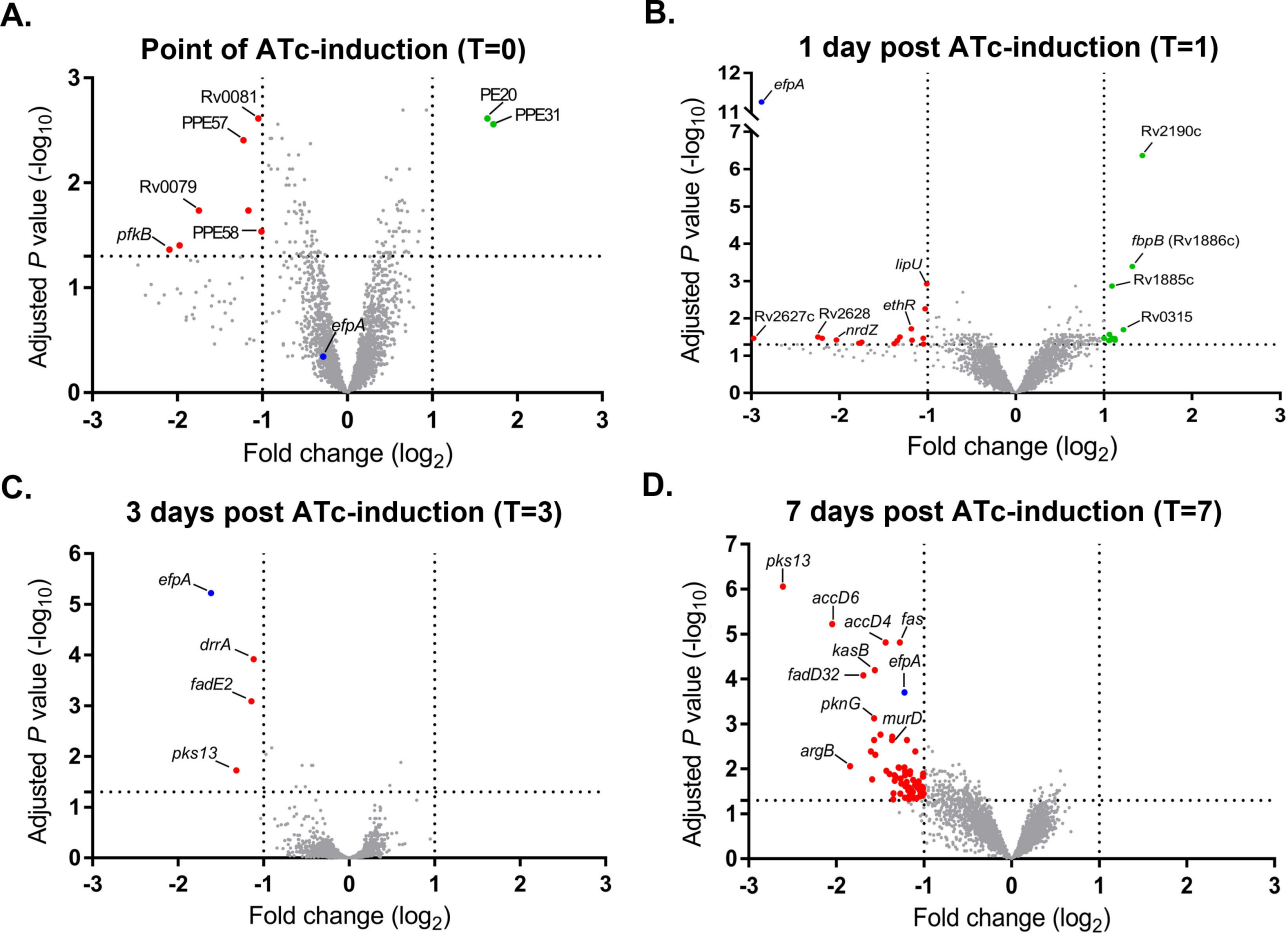
Volcano plots describing the *M. tuberculosis* transcriptional response to *efpA* repression. Expression data from three biological replicates were analyzed using a pipeline in Limma (30). Plots show the log_2_ fold-change in gene expression and log_10_-adjusted *P-*value for all genes in *efpA*^KD^ that were induced with 200 ng/mL ATc compared to no induction, at day 0 (**A**), day 1 (**B**), day 3 (**C**), and day 7 (**D**). Genes with increased expression are highlighted in green, and genes with reduced expression are highlighted in red. Significantly differentially regulated gene names are annotated where known. *efpA* is indicated in blue.

Whole-genome DNA microarray was used to identify pathways differentially expressed after *efpA* knockdown that could provide insights into the function of EfpA. The impact of *efpA* knockdown was observed over time, comparing e*fpA*^KD^ with or without ATc addition (Fig. 6). The raw data are available in ArrayExpress (ArrayExpress accession E-MTAB-13755), and the differentially expressed genes for all the timepoint comparisons are captured in the Supplementary Data (Excel file ”Differential_expression_efpA_knockdown _S1”). Reduced expression of *efpA* was observed in e*fpA*^KD^ at day 1 (−2.88-fold log_2_ repression), day 3 (−1.62-fold log_2_ repression), and day 7 (−1.22-fold log_2_ repression) post-ATc induction. During *efpA* repression, much of the differential gene expression (reduced expression only) was observed at day 7 (Fig. 6D) for functions (or putative functions) in DNA replication, cell division, redox/energetics, and cell wall lipid biosynthesis; our discussion will focus on these genes.

### Repression of *efpA* prevents regrowth of *M. tuberculosis* during INH exposure

**Fig 7:**
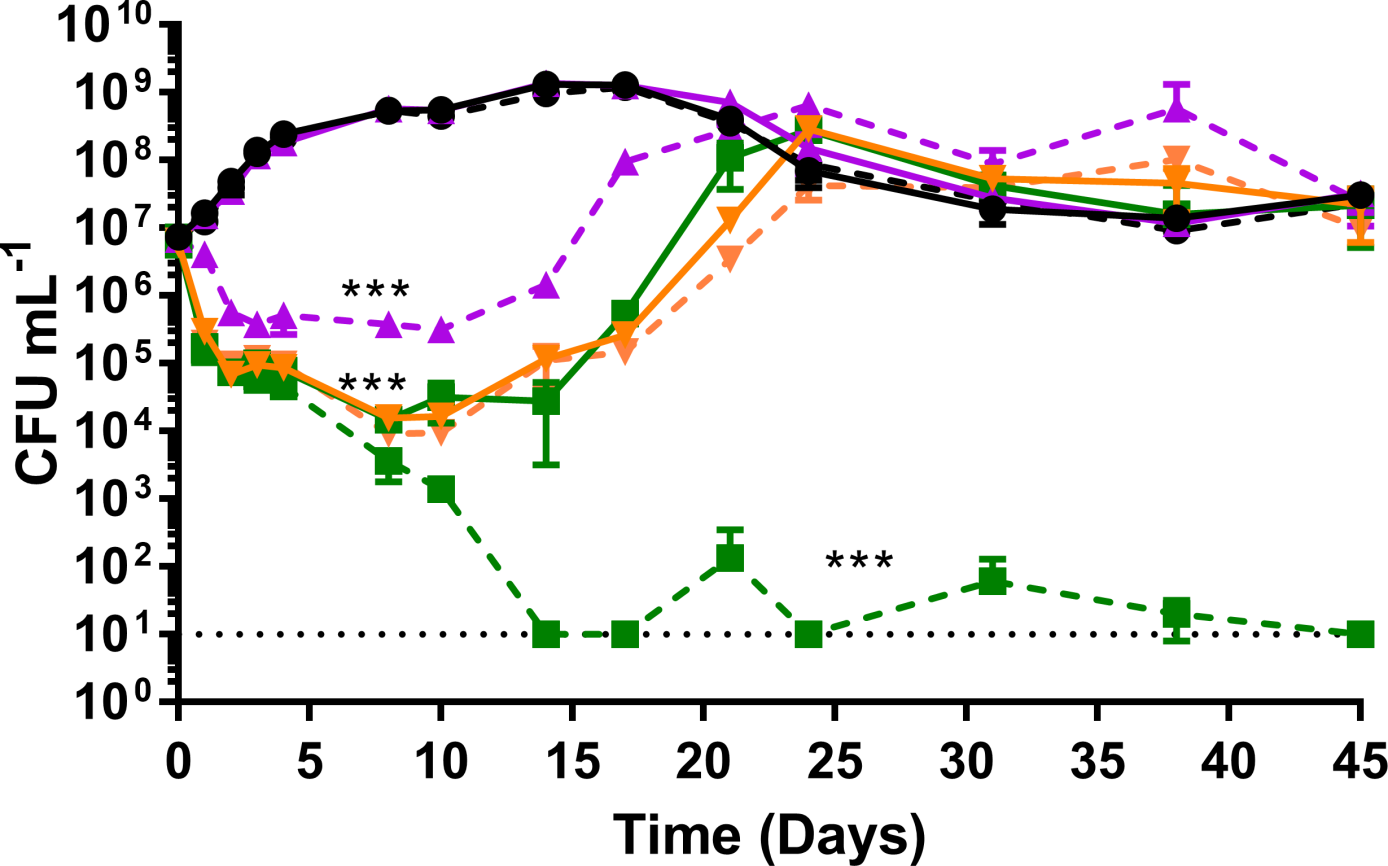
The impact of *efpA* repression on INH activity *in vitro*. The viability of the control strain (**solid lines**) and *efpA*^KD^ (**dotted lines**) over a 45-day time course, exposed to either 0.5 µg mL^−1^ of INH (**orange inverted triangles**), 200 µg mL^−1^ ATc (**purple triangles**), 0.5 µg mL^−1^ isoniazid and 200 ng mL^−1^ ATc in combination (**dark green squares**), or no drug (**black circles**). INH/ATc were added at culture inoculation (day 0). The *M. tuberculosis* viability was measured by viable counts (CFU mL^−1^). All values represent the mean of three biological replicates. Error bars are ±SD. A three-way ANOVA was used to compare viability (CFU mL^−1^) between strains with or without ATc induction and/or with or without INH-exposure. *** =*P* ≤ .001. Minimum CFU mL^−1^ values were set at the limit of detection (10 CFU mL^−1^) at days 14, 17, 24, and 45.

We determined whether *efpA* repression led to the increased sensitivity of *M. tuberculosis* to INH. The *efpA*^KD^ strain and the control strain were cultured for 45 days, in triplicate. At the point of inoculation, the following were added to cultures: either ATc at 200 ng mL^−1^, INH at 0.5 µg mL^−1^, a combination of INH/ATc, or no drugs at all. Culture samples were removed at days 0, 1, 2, 3, 4, 8, 10, 14, 17, 21, 24, 31, 38, and 45, for total viable counts (CFU mL^−1^). As expected, a bactericidal response was observed during INH exposure, which was not observed with ATc exposure. In the absence of INH, the ATc-induced *efpA*^KD^ cultures exhibited a reduction in viability due to *efpA* repression (Fig. 7), whereas the control strain grew normally, replicating our earlier findings (Fig. 2). In the presence of INH alone, both the *efpA*^KD^ and control strains demonstrated an equivalent steep drop in viability (3 logs_10_ CFU mL^−1^) over the first 3 days and started to recover by day 5, as expected from previously published *in vitro* INH exposure experiments (19, 21, 23). By day 24, both strains recovered to the same level of viability (CFU mL^−1^) as that prior to INH addition. The control strain exposed to a combination of ATc and INH followed the same triphasic pattern of killing, followed by recovery. For *efpA*^KD^, a combination of ATc and INH resulted in an initial decline in viability, which was consistent with the response to INH alone. However, between days 7 and 14, *efpA*^KD^ cultures exhibited a severe reduction in viability and fluctuated at a level of viability around the limit of detection (10 CFU mL^−1^) over the remainder of the 45-day time course (Fig. 7). In conclusion, the ability of *M. tuberculosis* to recover from INH exposure was abolished by the repression of *efpA*.

## DISCUSSION

### Targeted repression of *efpA* using CRISPRi results in a loss of viability in *M. tuberculosis*

EfpA is a putative efflux pump that may modulate the effectiveness of TB drugs. We used an inducible CRISPRi system to repress *efpA* expression in *M. tuberculosis* (26). This resulted in a statistically significant loss in the viability of *efpA*^KD^ and a triphasic response, that followed a similar trend to the response to INH; cidal, followed by static, and finally regrowth. This loss in viability during *efpA* repression can be explained by an initial loss of bacteria when *efpA* is fully repressed, followed by a phase of stalled growth during partial repression of *efpA,* and finally a period of recovery as the effects of ATc-induced *efpA* knockdown wane (Fig. 2 and 7 ). This profile is reflected in the levels of the *efpA* transcript over time. The *efpA* expression levels immediately decrease post-ATc addition and are at their lowest at day 1, upon which they start to increase. Despite this immediate decrease in *efpA*, the viability does not start to fall until day 2 post-exposure, which could be explained by the slow growth rate of *M. tuberculosis* and at least one to two doubling times required to see a population-wide effect. SEM provides supporting evidence for stalled replication (Fig. 4). Bacteria were observed with increased cell length, which appeared not to be dividing normally. Whole-genome transcriptome analyses highlighted reduced expression of genes at day 7 post-ATc induction, which supports a reduction in growth (ATP synthase: *atpD–atpF* and *atpH* and ribosomal proteins: *rpmD* and *rpmI*), cell division, (peptidoglycan biosynthesis: *murD*, *murF*, *murX*, and *ald*), and DNA replication and repair (reduction of ribonucleotides: *guaB1*, *nrdB, nrdI,* and *nrdH). Rv0232*, which encodes a Tet^R^-like regulator, which is colocalized (immediately upstream) and coexpressed with *nrdB,* was also reduced in its expression (36). Efflux pumps have previously been shown to be associated with Tet^R^ regulators (37). Another membrane protein, *mmpS5,* which effluxes bedaquiline, was also downregulated in *efpA*^KD,^ as was its repressor, *Rv0678* (38, 39). It would be interesting to see if either of the regulators described here control the expression of *efpA*.

### Repression of *efpA* prevented regrowth of *M. tuberculosis* during INH exposure

The focus of this study was to understand the significance of *efpA* in the susceptibility of *M. tuberculosis* to antibiotics, more specifically the recovery *in vitro* when the organism is exposed to INH. Repression of *efpA* combined with INH exposure caused a pronounced reduction in viability compared to INH alone and abolished the classic regrowth observed for *M. tuberculosis* when treated with INH *in vitro* (Fig. 7).

Regrowth after INH exposure has been observed in a variety of settings including *in vitro* batch log-phase cultures, continuous culture conditions, murine and guinea pig studies, and in patients with pulmonary tuberculosis (19, 21–24, 40, 41). We have previously sought to understand the reasons for this regrowth, using continuous culture, and addressed the hypothesis that regrowth was due to the persistence of drug-tolerant, slow-growing bacilli or the accumulation of INH drug-resistant mutants. We found through a combination of two studies that regrowth occurred irrespective of growth rate, INH concentration, or INH in combination with other antibiotics. INH resistance mutations only partially explained regrowth (with INH singly or in combination) (19, 23). Transcriptomics studies highlighted a variety of potential tolerance mechanisms, including upregulation of efflux pumps (19). We observed, using chemostat culture, that *efpA* was more highly expressed during the early bactericidal activity (EBA), at 2 days post-INH addition (19). In the current study, *efpA* repression led to a longer bactericidal phase as opposed to an increase in the rate of INH-induced killing. Given the upregulation of *efpA* observed during EBA in chemostats, it seems likely that *efpA* has a role in enabling the bacilli to survive and recover. Repression of *efpA* could lead to a reduction in the level of INH expelled from the cell, resulting in increased INH intracellular concentrations, thereby halting regrowth. In 2012, Schmalstieg et al. (25) postulated that efflux pump induction could lead to development of resistance-associated mutations. Alternative explanations could be that cell wall modifications observed after *efpA* repression may enhance the activity of INH. The transcriptomic analyses at day 7 after ATc addition and in the absence of INH indicated changes in cell wall processes and more specifically alterations in the biosynthesis of mycolic acids or their precursors (*kasA, kasB, accD6, accA1, fas, accD4, pks13,* and *fadD32*). INH targets the FAS-II biosynthesis pathway; therefore, a lower expression of these enzymes during *efpA* repression may be responsible for potentiating the activity of INH.

### Is EfpA a good target for therapeutics?

This study provides further evidence that EfpA is essential in *M. tuberculosis* and may be a legitimate target for drug discovery. Our finding that repression of *efpA* prevents regrowth of *M. tuberculosis* after INH exposure is significant, especially for retaining the use of INH in current TB regimens. However, translating these *in vitro* results to *in vivo* infection will be challenging. In the first instance, it will be important to see how the inhibition of EfpA impacts INH efficacy in animal models of *M. tuberculosis* infection and ultimately whether relapse is reduced. The early bactericidal activity of INH reduces the bacterial burden in TB patient sputa substantially over the first few days of therapy; if this killing could be enhanced by an efflux inhibitor, then the use of INH for treatment of susceptible TB could be extended. EfpA was characterized in a recent chemical genetic screen (8, 42), which resulted in the development of two inhibitors that exhibited good activity against EfpA: BRD-8000.3 and BRD-9327. Further studies will determine whether the inhibition of EfpA is a viable strategy to augment multidrug therapy for tuberculosis by repressing efflux activities. These investigations would include experiments to understand the interplay between EfpA repression and the activity of other antibiotics in addition to INH.

## Data Availability

UKHSA has an open-access policy; all supplementary data will be available online with the published article. Microarray data are available through Array Express, accession number E-MTAB-13755.

## SUPPLEMENTAL MATERIAL

Supplemental material aac.00261-24-s0001.xlsx Tables displaying the log_2_ fold changes in gene expression and -log_10_ adjusted P-values for genes in *M. tuberculosis efpA*^KD^ that has been induced with 200 ng mL ^-1^ ATc compared to *M. tuberculosis efpA*^KD^ without ATc induction, at days 0, 1, 3, and 7.

ASM does not own the copyrights to Supplemental Material that may be linked to, or accessed through, an article. The authors have granted ASM a non-exclusive, world-wide license to publish the Supplemental Material files. Please contact the corresponding author directly for reuse.
